# Antibacterial Properties of a Honeycomb-like Pattern with Cellulose Acetate and Silver Nanoparticles

**DOI:** 10.3390/ma14144051

**Published:** 2021-07-20

**Authors:** Klaudia Hurtuková, Klára Fajstavrová, Silvie Rimpelová, Barbora Vokatá, Dominik Fajstavr, Nikola Slepičková Kasálková, Jakub Siegel, Václav Švorčík, Petr Slepička

**Affiliations:** 1 Department of Solid State Engineering, University of Chemistry and Technology Prague, Technická 3, 166 28 Prague, Czech Republic; klaudia.hurtukova@vscht.cz (K.H.); klara.fajstavrova@vscht.cz (K.F.); dominik.fajstavr@vscht.cz (D.F.); nikola.kasalkova@vscht.cz (N.S.K.); jakub.siegel@vscht.cz (J.S.); vaclav.svorcik@vscht.cz (V.Š.); 2 Department of Biochemistry and Microbiology, University of Chemistry and Technology Prague, Technická 3, 166 28 Prague, Czech Republic; barbora.vokata@vscht.cz

**Keywords:** biopolymers, honeycomb-like pattern, surface nanostructures, silver nanoparticles, antimicrobial activity, surface morphology, antibacterial properties, silver sputtering, active materials

## Abstract

This study involved the preparation and characterization of structures with a honeycomb-like pattern (HCP) formed using the phase separation method using a solution mixture of chloroform and methanol together with cellulose acetate. Fluorinated ethylene propylene modified by plasma treatment was used as a suitable substrate for the formation of the HCP structures. Further, we modified the HCP structures using silver sputtering (discontinuous Ag nanoparticles) or by adding Ag nanoparticles in PEG into the cellulose acetate solution. The material morphology was then determined using atomic force microscopy (AFM) and scanning electron microscopy (SEM), while the material surface chemistry was studied using energy dispersive spectroscopy (EDS) and wettability was analyzed with goniometry. The AFM and SEM results revealed that the surface morphology of pristine HCP with hexagonal pores changed after additional sample modification with Ag, both via the addition of nanoparticles and sputtering, accompanied with an increase in the roughness of the PEG-doped samples, which was caused by the high molecular weight of PEG and its gel-like structure. The highest amount (approx. 25 at %) of fluorine was detected using the EDS method on the sample with an HCP-like structure, while the lowest amount (0.08%) was measured on the PEG + Ag sample, which revealed the covering of the substrate with biopolymer (the greater fluorine extent means more of the fluorinated substrate is exposed). As expected, the thickness of the Ag layer on the HCP surface depended on the length of sputtering (either 150 s or 500 s). The sputtering times for Ag (150 s and 500 s) corresponded to layers with heights of about 8 nm (3.9 at % of Ag) and 22 nm (10.8 at % of Ag), respectively. In addition, we evaluated the antibacterial potential of the prepared substrate using two bacterial strains, one Gram-positive of *S. epidermidis* and one Gram-negative of *E. coli*. The most effective method for the construction of antibacterial surfaces was determined to be sputtering (150 s) of a silver nanolayer onto a HCP-like cellulose structure, which proved to have excellent antibacterial properties against both G+ and G− bacterial strains.

## 1. Introduction

Natural patterns and structures provide inspiration for scientists of diverse technological backgrounds to create artificial products (from different materials) with similar properties as naturally occurring products [1,2]. One such pattern is the naturally occurring honeycomb-like pattern (HCP) [2,3]. The surfaces of products with this pattern consists of thousands of interconnected hexagonally formed cells that create an efficient structure with a large surface area. The HCP, due to its excellent properties, such as structural and mechanical strength, low density, and porosity, has found applications in several areas, including architecture, chemical engineering, mechanical engineering, and biomedicine [1,2,4]. HCP-like structures have also been widely used as carriers in tissue engineering (TE) [5].

Carriers that are mainly used in TE should mimic their extracellular matrix (ECM) morphology to ensure compatibility with living organisms and a three-dimensional (3D) structure. The ECM provides a natural environment for cultured cells, improving their adhesion, proliferation, and differentiation [6,7,8]. The surface morphology and porous nature of HCP-like structures make them irreplaceable substrates that are useful for cell differentiation and proliferation and the creation of functional and protective sites for the adhesion of biomolecules and growth factors and the production of specific drug delivery spaces [9]. A suitable alternative to carriers for different 3D cell cultures appears to be HCP-like film structures due to their geometric regularity, which can provide mechanical and biochemical advantages in the ECM, as in living organisms [10].

The HCP-like films can be prepared in two ways, namely the (i) breath figure (BF) and (ii) improved phase separation (IPS) approaches. In 1994, Widawski et al. first prepared HCP-like films [9,11] and found that factors such as the wet conditions, solvent type, polymer structure, and molecular weight affect the spontaneous organization of pores in periodic hexagonal fields [9]. This method was named the BF approach and has undergone substantial evolution in recent decades [12]. This method has attracted that attention of scientists, mainly due to its simplicity (few steps and low complexity of preparation), economic feasibility, use of harmless and affordable media (water), fast preparation times for many porous films with large surface areas, and applicability to various polymers, as well as this method allowing the tailoring of the size and shape of the pores by changing the process parameters, such as the air humidity and polymer concentration [9]. Although the BF method has many of the mentioned positive properties in terms of versatility and cost-effectiveness, it may have applicability for commercial polymers and in very humid process conditions [13].

To avoid high humidity, low volatility solvents can be added to the polymer solution. The IPS method uses a two-step film-forming process that can be used on many commercially available polymers. In this procedure, methanol (MeOH) is added directly to the polymer solution in the chloroform (CHCl_3_) to form a tertiary polymer–good solvent–bad solvent system [13,14]. The ordered HCP-like structures on the surfaces of the substrates are formed after immersion of the sample into the polymer solution and subsequent drying in normal ambient air without adding additional moisture. The surface morphology of the structures depends mainly on the amount of MeOH added, but also on the concentration of the prepared solution and the ambient humidity; however, with the IPS method, the key factor affecting the pore shape, size, number, and density is the volume of MeOH in the solution. With a low MeOH content in the solution (below 10%, *v*/*v*), small round pores can be detected on the surface of the polymer. At the concentration of 15% (*v*/*v*), the pores have a hexagonal shape and are close to each other. In the case of a surplus or without the addition of methanol, the HCP-like structures are disrupted [13,14].

The cellulose acetate polymer is the most interesting of the cellulose derivatives for a wide variety of applications [15,16]. Due to its properties, including its relatively low cost, biocompatibility, biodegradation in human and animals [16,17], nonpoisonousness, mechanical strength, and dissolution in water, cellulose acetate has mainly been utilized in the field of TE [15,17,18]. It has also found use in bioapplications, drug delivery, antibacterial applications, and wound dressings [16,18,19]. Acetylation of cellulose reduces its crystallinity, providing improved biodegradability in vivo compared to plant cellulose and some of its derivatives. Ester and aerobic conditions also promote degradation [18,20,21]. IPS methods can be used with cellulose acetate to form regular hexagonal HCP-like structures.

In the search for a polymer with CA-like properties, the non-biodegradable polyethylene glycol (PEG) polymer has proven to be a suitable candidate. Comparable to CA, PEG is used mainly as a carrier, including for drug delivery or for applications involving organs and tissues [22,23]. Additionally, it is resistant to protein absorption, making it suitable for in vivo and in vitro studies [24]. PEG is mostly used in the form of hydrogels. Its properties imitate a three-dimensional environment similar to soft tissues and enable the diffusion of nutrients and cell waste [25,26]. PEG is a biodegradable polymer only when copolymerized with other biodegradable polymers, such as polyglycolic acid (PGA) and poly-L-lactide acid (PLA) [27]. Many scientists have reported that PEG-based surfaces offer protection from external contamination; however, the protection level is not very high and a certain number of bacteria can get onto the polymer [28,29,30,31]. This is why the main goal is to find an antibacterial agent that can efficiently eliminate bacterial contamination, while at the same time being biocompatible with the human body [32]. One such effective option is silver nanoparticles (AgNPs), since they have good antibacterial, antiviral, and antifungal activity [33,34,35,36]. Ag acts as an antibacterial agent in its ionic form at low concentrations, although no significant antibacterial effect was found in the Ag^0^ form. The deposition of an Ag layer on a substrate’s surface is mostly achieved by sputtering in a vacuum environment [37].

In this study, we focused on the effects of Ag nanoparticles sputtered on the surfaces of HCP-like structures or incorporated into their morphology. The changes of the surface morphology of the modified structures, their chemical compositions, and their antibacterial effects were investigated and compared with an unmodified sample with HCP-like structures. The effects of the combination of both aspects increased the effective surface area of the HCP-like pattern, while the nanocluster surface formation effectively inhibited the growth of the selected bacteria. To the best of our knowledge, the antibacterial properties of the silver-sputtered HCP cellulose acetate pattern have not been reported to date.

## 2. Results

### 2.1. Surface Morphology and Roughness

**Figure 1:**
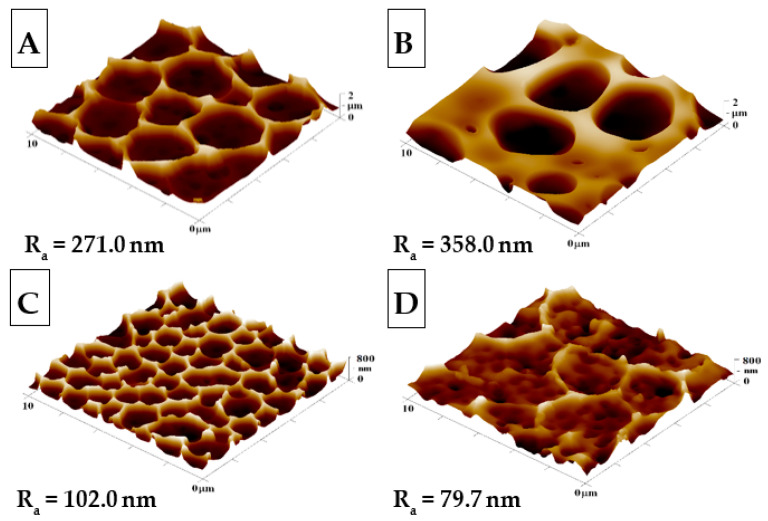
The surface morphologies of the prepared samples: (**A**) honeycomb-like (HCP) structure of the plasma-treated fluorinated ethylene propylene (FEP); (**B**) HCP structure with built-in polyethylene glycol (PEG) with sputtered Ag nanoparticles; (**C**,**D**) HCP structures with Ag nanoparticles sputtered for 150 and 500 s. The inspected area measured 10 × 10 µm^2^. R_a_ represents the average roughness in nm.

The goal of this study was to create an HCP-like pattern on FEP polymers and to modify these structures with Ag. Modification by plasma discharge can substantially affect the chemical composition and structure of a material; therefore, the criteria for substrate modification are significant for the resulting polymer surface [38]. The most suitable parameters for plasma etching were selected according to the study reported by Neznalová et al. [39], i.e., material modification at 8 W for 240 s, in order to create a suitable surface for HCP-like structures. The modified honeycomb pattern was prepared by sputtering Ag nanoparticles onto the HCP-like pattern (Figure 1C,D) or by incorporating them into PEG. This mix was added to the solution for preparation of the HCP-like structures (Figure 1B).

The surface morphology, roughness, and effective surface area for HCP and modified HCP structures with Ag were determined using the AFM method (see Figure 1). From Figure 1, we can see that the “pristine” HCP structure (A) contains hexagonal pores formed on its surface using the IPS method, with slight variations from the optimal hexagonal pattern; however, in comparison to other samples, it exhibited the most representative HCP-like structure without any further modification. After additional sample modification with Ag (Figure 1B–D), one can see the change from the optimal HCP-like pattern, which was mostly caused by the presence of Ag deposited in PEG added into the source polymer solution (Figure 1B). PEG has a high molecular weight and a gel-like structure, which may have resulted in the increase in the roughness of sample B to 358.0 nm when compared to unmodified sample A, with a roughness of 271.0 nm. The second variation of the honeycomb pattern was achieved through direct sputtering on the surface with the HCP-like structure (Figure 1C,D). The AFM scans in Figure 1 show Ag nanostructures and their reduced roughness, which were created during the before mentioned process (Ag thin layer). In sample C, the HCP-like structure was destroyed through the disintegration of the walls, while the “hexagonal” pores formed had smaller diameters and heights than unmodified sample A. Comparing the sputtered sample (Figure 1D) with the unmodified sample (Figure 1A), we observed a significant decrease in pattern homogeneity. The difference was confirmed repeatably, which may have been caused by either the impacts of silver atoms with high energy filling the cavities with silver nanoclusters; however, this difference in morphology was still surprising.

### 2.2. Surface Morphology Analysis Using SEM and Surface Chemistry Analysis

**Figure 2:**
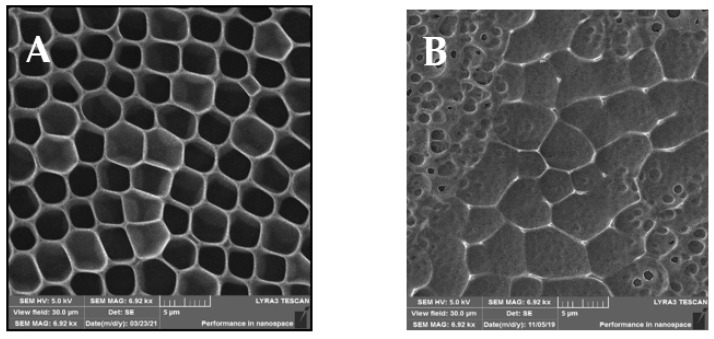
SEM scans of the prepared samples: (**A**) a honeycomb-like (HCP) structure on the plasma-treated fluorinated ethylene propylene (FEP); (**B**) HCP structures with silver nanoparticles sputtered for 150 s. The inspected area was 30 × 30 µm^2^.

The SEM analysis of the prepared samples is depicted in Figure 2, showing different views of the morphologies of the prepared samples. Compared to the AFM scans in Figure 1, in the SEM scans, the unmodified sample A indicates a structure with an expressive hexagonal shape. New arrays were formed between the “hexagonal” structure, which can be observed on the modified sample B. After sputtering of AgNPs, small spherical pores were created. On the other hand, similar HCP structure destruction can be seen, especially in their walls, in comparison to the AFM scans. The destruction and new pores may have been formed by the high rate of sputtered AgNPs from the target onto the structure.

**Figure 3:**
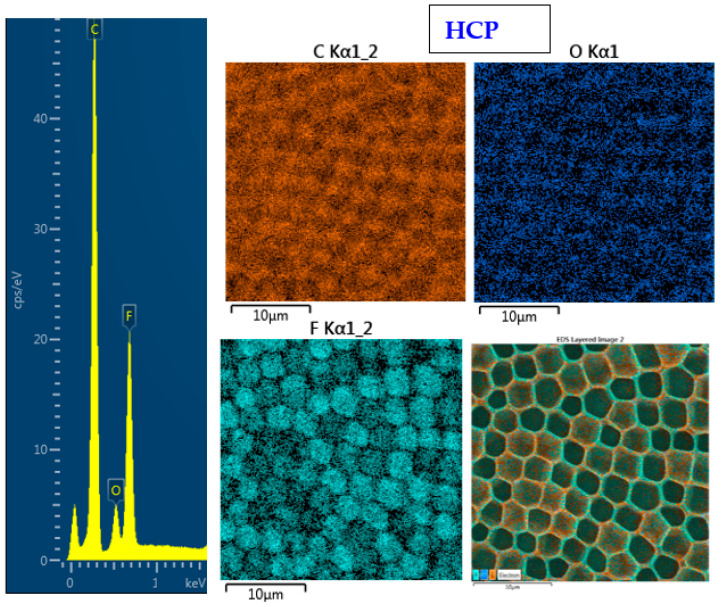
EDS spectrum and elemental maps with layered EDS images for the honeycomb-like (HCP) structure on the plasma-treated fluorinated ethylene propylene (FEP).

**Figure 4:**
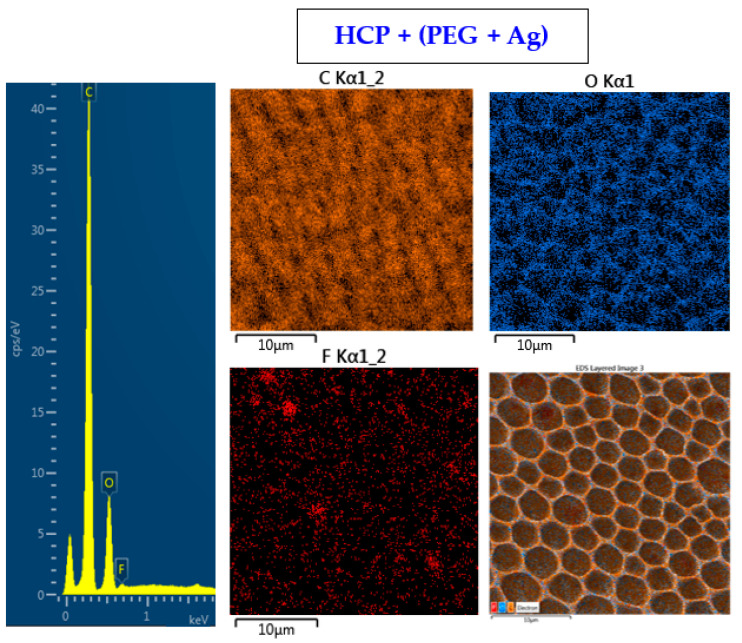
EDS spectrum and elemental maps with layered EDS images for the honeycomb-like (HCP) structure containing polyethylene glycol (PEG) with sputtered Ag on the plasma-treated fluorinated ethylene propylene (FEP).

**Figure 5:**
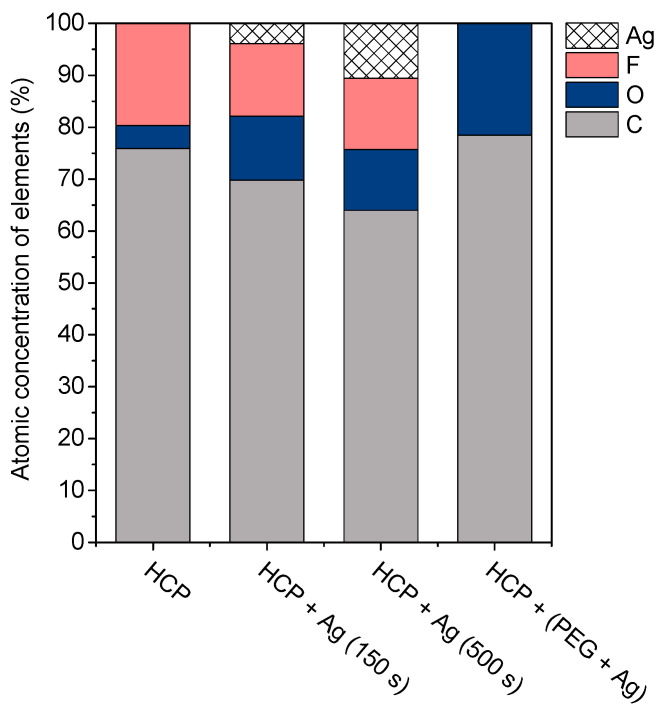
The atomic concentrations of Ag, F, O, and C elements (in at %) were determined using the energy-dispersive X-ray spectroscopy method for the sample with a honeycomb-like (HCP) structure, samples with Ag thin layers with the different time lengths of metal deposition (150 s and 500 s), and the sample containing polyethylene glycol (PEG) with sputtered Ag.

The surface chemistry of the prepared samples with the HCP-like structure was determined by EDS analysis and the results are shown in Figure 3, Figure 4 and Figure 5. For the EDS method, the spatial resolution ranges from 50 nm to 1 μm [40]. The main characteristic of the FEP polymer is the large amount of fluorine introduced in its structure. This meant that all samples (except the sample with PEG + Ag) containing the fluorine element were measured through the structure up to the substrate polymer. The highest amount (approximately 25 at %) of fluorine was detected on the sample with an HCP-like structure, while the lowest amount (0.08%) was measured on the PEG + Ag sample. Samples with Ag layers (150 and 500 s) had similar fluorine concentrations of approximately 14%. As expected, the thickness of an Ag layer on the HCP surface depended on the length of sputtering (either 150 s or 500 s) of Ag. The sputtering time for Ag (150 s and 500 s) corresponded to layers with heights of about 8 nm (3.9 at % of Ag) and 22 nm (10.8 at % of Ag), respectively. The sample with PEG incorporated in the structure did not reveal the presence of AgNPs, which may have been caused by AgNPs penetration deeper into the bulk of the material. On the other hand, the sample with PEG + Ag had the highest oxygen concentration (21.4%). This was probably caused by the presence of PEG in the biopolymer foil and the content of oxygen atoms in its natural structure. The fluorine was also not detected, since even in the “lower” area (with lower thickness) of the honeycomb-like unit, the thickness of it was still higher than approximately 100 nm; therefore, it was able to “shield” the fluorine signal from the substrate below. We can deduce from the EDS analysis that AgNPs bound with oxygen were located in the atmosphere and according to the study by Thijssen et al., who proved that oxygen atoms can be incorporated into atomic chains with noble atoms [41]. This effect may be the reason for the higher oxygen concentrations (around 12%) for samples with a thin Ag layer. The lowest oxygen concentration (4.5%) was detected on the surface of the sample containing HCP.

**Figure 6:**
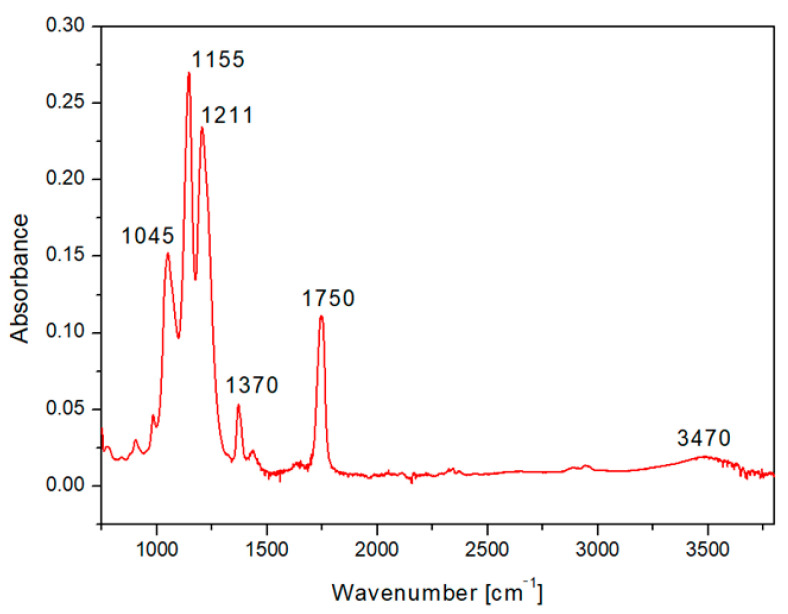
The FTIR spectrum of fluorinated ethylene propylene (FEP) with the prepared honeycomb-like (HCP) structure from acetate cellulose and with an Ag layer sputtered for 500 s.

We selected for presentation the typical HCP pattern sputtered with 500 s Ag (Figure 6). The FTIR spectrum of the CA honeycomb material covered with Ag showed the characteristic bands attributed to the vibrations of the acetate group: the carbonyl stretching at 1750 cm^−1^ (νC=O), methyl bending at 1370 cm^−1^ (δC-CH3), and peak at 1211 cm^−1^, attributed to C–O stretching of the acetyl group. A strong band at 1155 cm^−1^ (due to C–O antisymmetric bridge stretching and C–O–C pyranose ring skeletal vibration) was also detected, as well as a band at 1045 cm^−1^ (C–O–C stretching of pyranose ring), while the broad hydroxyl group absorption appeared at approximately 3470 cm^−1^. The results indicated that even after sputtering of the acetate cellulose honeycomb pattern, the spectrum was in good accordance with typical acetate cellulose spectra presented in [42,43].

### 2.3. Wettability

**Figure 7:**
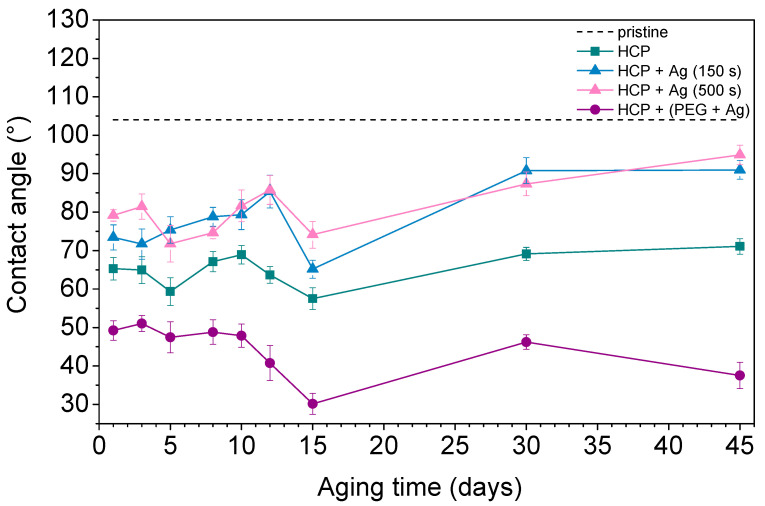
The water contact angles over 45 days for pristine fluorinated ethylene propylene (FEP) and the prepared honeycomb-like (HCP) structure and its modified samples, with Ag layers sputtered for 150 s and 500 s and deposition of AgNPs into polyethylene glycol (PEG).

Contact angle measurement is one of the effective methods used to understand surface properties such as wettability, adhesion, and surface energy. A hydrophobic surface (high contact angle) indicates poor sample wettability, while a hydrophilic surface (low contact angle) indicates better physical properties [44]. Figure 7 shows the contact angles of the prepared FEP samples as well as pristine FEP over the 45 days that the aging study was performed. The contact angle on pristine FEP was determined to be 104° based on further studies. Additionally, other samples underwent changes in their surface wettability during the aging study. The plasma treatment used for the sample preparation decreased the contact angle by creating radicals on the surfaces and reactions leading to oxygen-containing groups. PEG also contains hydroxyl groups in its structure, which give it a more hydrophilic surface compared to pristine biopolymer while having the lowest contact angles (ranging from 30° to 50°) during the aging process. After 45 days, structures with thin Ag layers (150 s and 500 s) on the surface had very similar contact angles (approx. 90°). Their surfaces were more hydrophobic than other prepared samples however, they still had more oxygen in their structures (see Figure 6, HCP + Ag 150 s/+ Ag 500 s). This observation indicated certain differences between the contact angle measurements (sample surface, approx. top ten atomic layers) and the EDS treatment (in-depth analysis). The possible reason may be that AgNPs with bound oxygen rotate into the structure or migrate deeper into polymer chains. The HCP sample maintained a mean value of about 70°. It can be concluded that the contact angle depends on the physicochemical properties rather than on the surface structure. The lowest contact angles were observed in samples with silver nanoparticles incorporated in combination with oxygen-containing PEG chains, which were maintained during the aging process over 45 days.

### 2.4. Antibacterial Properties

**Figure 8:**
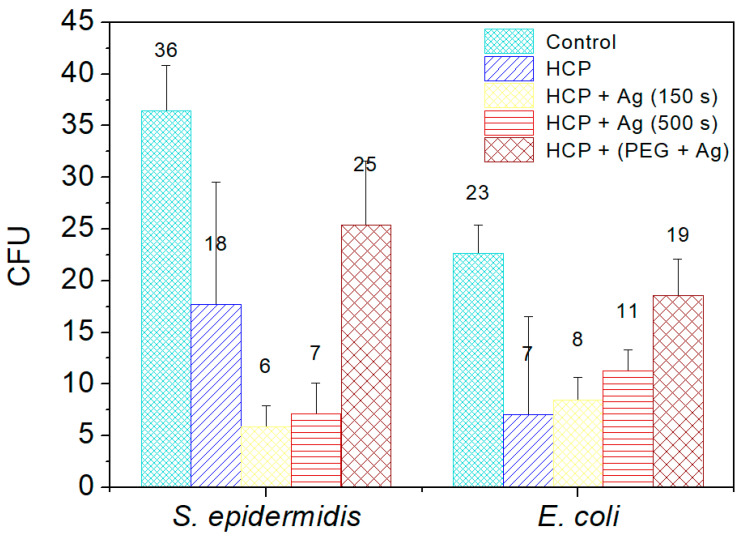
The numbers of colony-forming units (CFU) of *S. epidermidis* and *E. coli*, which were applied on the surfaces of the prepared samples of fluorinated ethylene propylene (FEP) for 2 h. The samples were tested after each phase of preparation: (1) honeycomb-like (HCP) structure formation; (2) sputtering of Ag layers for 150 s and 500 s on the HCP surface; (3) sputtering of Ag into polyethylene glycol (PEG) and embedding of the mixture into a solution in the HCP structure. Bacterial suspension incubated only with phosphate-buffered saline without any sample addition served as a control. The samples were done in triplicate.

Two commonly occurring bacterial strains were chosen as model microorganisms in order to evaluate the antibacterial activity of the prepared nano- and microstructures; specifically, these were Gram-negative (G−) *E. coli* and Gram-positive (G+) *S. epidermidis*. In this study, we aimed to identify the antibacterial properties of HCP-sputtered AgNP structures and samples with Ag incorporated into the polymer bulk against selected G+ and G− bacteria. Figure 8 represents the average numbers of colony-forming units for both bacterial strains incubated in the selected samples.

Ag^+^ in a monoatomic–ionic state created through the oxidative dissolution of the Ag^0^ NPs on the HCP surface was the appropriate antibacterial agent (see results in Figure 8) [45]. Differences occurred in the sample with AgNPs sputtered into PEG. The results of the EDS analysis showed no Ag elements in the sample structure and induced a low level of bacterial inhibition. PEG has antibacterial properties by itself, but is not as effective as ionic Ag^+^. Vasudevan et al. designed patterned structures in different sizes and observed that all prepared patterned surfaces were covered with a considerably low number of bacterial colony-forming units compared to flat surfaces [46]. In the same way, we prepared an excellent antibacterial patterned structure and were able to detect antibacterial activity on the modified HCP-like structure. Regarding the antibacterial activity of nanostructured Ag, the interaction is usually based on two synergistic processes. The first one is connected with the direct contact of the bacterium with noble metal Ag, while the second effect, which influences the bacteria, is the release of Ag^+^ ions into the medium and their subsequent interaction with the bacteria, depending on the specific conditions [45,46,47,48]. Ag ions may interact with four major components of cell bacteria, which are the plasma membranes, cell walls, bacterial DNA, and proteins. For our study, we assumed that the antibacterial activity of *E. coli* is based on the direct interactions of Ag atoms with bacteria and partially on the release of Ag^+^ into the environment, as both processes take place. The Ag^+^ also causes degradation of the peptidoglycan cell wall leading to cell lysis (cell death), preventing further proliferation of bacteria. The ions may also penetrate into the inner part of the bacteria and are bound on the basis of their DNA.

## 3. Materials and Methods

### 3.1. Materials

In this study, we used acetate cellulose (CA, powder form, M_r_ = 300,000 g·mol^−1^, density of 1.3 g·cm^−3^; purchased from Sigma-Aldrich, St. Louis, MO, USA) and PEG (liquid medium for the preparation of a colloidal solution of silver nanoparticles, Mr = 600 g·mol^−1^, density of 1.13 g·cm^−3^; purchased from Sigma-Aldrich, St. Louis, MO, USA). As the solvents, we used methanol (MeOH, for HPLC, Mr = 32.04 g·mol^−1^, density of 0.791 g·cm^−3^; purchased from Penta, Prague, Czech Republic) and chloroform (CHCl_3_, stabilizer with ~1% ethanol A.G., M_r_ = 119.38 g·mol^−1^, density of 1.48 g·cm^−3^; purchased from Penta, Prague, Czech Republic). As a substrate, we used fluorinated ethylene propylene polymer (FEP, thickness of 50 mm, density of 2.15 g·cm^−3^; purchased from Goodfellow Ltd., Cambridge, Huntingdon, UK). A silver target (purity of 99.999%; purchased from Safina, Vestec, Czech Republic) was used to prepare the nanoparticles. The substances used in antibacterial tests were Luria–Bertani (LB) liquid medium for culturing bacteria and phosphate-buffered saline (PBS).

### 3.2. Pattern Preparation and Modification

The polymer film was treated by Ar^+^ plasma using the SCD 050 sputtering device from BAL-TEC. The purity of gas in the chamber was 99.997% and the pressure was 8 Pa. The samples were placed on a circular holder (anode) with a diameter of 10 cm and a distance of 5 cm from the cathode. They were modified at 8 W for 240 s.

In the next step, HCP-like structures were formed using the improved phase separation method via immersion of plasma-modified polymeric substrates in the prepared 100 mL solution for 10 s. The solution was a mixture of two solvents, namely chloroform and methanol, at a volume ratio of 85:15. Then, 2 g of cellulose acetate was added to the mixture while stirring, which gave a homogeneous solution. Then, the HCP-like structures were removed and left to air dry in Petri dishes. After complete evaporation of the solvents, the samples were prepared for further modification and were also subjected to examination using various analytical methods.

### 3.3. Silver Nanostructure Preparation

Sputtering of thin Ag layers on the surfaces of the prepared HCP-like structures was performed using the Quorum Q300T ES with cathodic sputtering. Different sputtering times were used (150 s and 500 s), with a constant sputtering current of 20 mA. A set of samples was prepared to determine the thicknesses of the sputtered thin Ag layers using the scratch test method. Ag layers were deposited on glass slides, while the experimental conditions were the same as in the previous case for the deposition of Ag onto HCP-like structures.

Sputtering of Ag nanoparticles into 2 mL of PEG solution was performed using a Quorum Q 150RS instrument. The deposition time was 300 s and the current was 30 mA. The concentration of Ag nanoparticles in 2 mL of PEG was 1.05 mg·mL^−1^. Subsequently, the solution was added to the polymer solution (CHCl_3_, MeOH, CA). We determined the size of the gold nanoparticles to be 8 nm [49].

We investigated the differences between the surface morphologies of the samples including an unmodified substrate (pristine), a substrate with HCP-like structures (HCP), a substrate with sputtered Ag layers on the surfaces of HCP-like structures (HCP Ag; layers sputtered for 150 s and 500 s), and a substrate with Ag nanoparticles sputtered into the PEG and subsequent incorporation into HCP structures (HCP (PEG + Ag)).

### 3.4. Analytical Methods

The wettability of all samples was studied via goniometric measurements of the contact angles, with a drop of distilled water applied to the surface of each sample (6 positions). The contact angles of the samples were determined using a See System goniometer (Advex Instruments) at room temperature. A drop of water (8 μL) was applied onto each sample with a Transferpette^®^ automatic pipette (Brand, Wertheim, Germany).

Atomic force microscopy (AFM) was used to study the surface morphology of the FEP substrate with HCP microstructures. The movement of the tip as it passed over the sample was recorded and a point-by-point image of the surface was compiled. A Dimension ICON atomic force microscope with a SCANASYST-AIR Si tip from Bruker Corp. (Billerica, MA, USA) was used for the measurement. At the same time, the mean surface roughness (R_a_) was determined, which represents the arithmetic mean of the absolute values of the height deviations measured from the central plane. Samples were measured in tapping mode (RTESPA probe with constant elasticity 40 N m^−1^) or QNM (Scan Asyst air probe, elasticity constant of 0.4 N m^−1^). The thicknesses of the sputtered Ag layers on glass samples were also measured using the scratch test and subsequent AFM analysis.

Scanning electron microscopy (SEM) (Tescan, Brno, Czech Republic) and energy-dispersive X-ray spectroscopy (EDS) were used for detailed analysis of the morphology and chemical characterization of the FEP substrate with HCP microstructures. We used a scanning electron microscope LYRA3 GMU (Tescan, Brno, Czech Republic) with accelerating voltage of 10 kV for the electrons that bombarded the samples and an F-MaxN analyzer and SDD detector (Oxford Instruments, Abingdon, UK) with an applied accelerating voltage of 10 kV for EDS. Platinum (target, purity 99.999%, Safina, Vestec, Czech Republic) was sputtered onto the samples before analysis using a Quorum Q300T sputtering device at a current of 30 mA for 400 s.

The FTIR system we used was a Nicolet iS5 (Fisher Scientific, Waltham, MA, USA) with a diamond crystal iD7 ATR accessory. The spectra were obtained as averages from 128 measurement cycles in the 4000–600 cm^−1^ spectral range with 0.964 cm^−1^ data intervals. An atmospheric suppression feature was employed to eliminate ambient CO_2_ and H_2_O concentration changes.

### 3.5. Antibacterial Tests

Gram-negative and Gram-positive bacterial strains of *E. coli* (DBM 3138) and *S. epidermidis* (DBM 2124), respectively, were used for the evaluation of antibacterial tests of the prepared HCP-like structures. The bacterial strains were transferred from stock agar plates into LB liquid medium and incubated at 37 °C over 24 h while gently shaking. Bacterial growth was confirmed by measuring the optical density (OD) at 600 nm. Subsequently, the bacterial suspensions were diluted in prewarmed (37 °C) PBS. Then, 125 μL of the bacterial suspension with PBS was applied on the surface of each sample. Five 25 μL drops of bacterial suspension from each sample were then loaded onto LB and PCA agar plates used for *E. coli* and *S. epidermidis*, respectively. The samples were then cultured overnight at 37 °C. The tests were performed on three samples from each preparation step, which means that there were 15 drops for one preparation phase. At the same time, a control was performed by applying 15 drops of the bacterial suspension incubated only in PBS (with no sample added) onto agar plates, which were then treated in the same way as the samples.

## 4. Conclusions

We prepared an HCP-like patterned structure with sputtered AgNPs on the plasma-activated surface of an FEP polymer. The Ag nanostructure was prepared in two forms—as a thin layer on the HCP-like surface or sputtered into PEG, which was used for HCP preparation itself. Through combinations of the proposed modification methods (plasma exposure, addition of AgNPs into the source solution, direct Ag deposition, and isolated cluster formation), we managed to prepare HCP-like structures with differences in morphology, surface chemistry, wettability, and antibacterial properties. The plasma deposition process created an optimal surface for the formation of an HCP-like cellulose acetate structure. The HCP samples also had good surface wettability, and surprisingly the HCP-like pattern from cellulose acetate significantly suppressed the colonization of both *S. epidermidis* and *E. coli*. Sputtering of thin Ag layers increased the contact angle of the pattern, causing particular disruption but combined with remarkable effects against both evaluated bacterial strains. The greatest decreases of CFU for both bacterial strains were determined for HCP-like units sputtered with Ag for only 150 s. The incorporation of AgNPs into the polymer solution with PEG also decreased the uniformity of the HCP pattern. The selected samples are good candidates for testing in vitro for scaffold applications in tissue engineering.

## Data Availability

Not applicable.

## Abbreviations

3D	Three-dimensional
AFM	Atomic force microscopy
BF	Breath figure
CFU	Colony-forming units
ECM	Extracellular matrix
EDS	Energy-dispersive X-ray spectroscopy
HCP	Honeycomb pattern
LB	Luria–Bertani
IPS	Improved phase separation
PBS	Phosphate-buffered saline
PCA	Plate counting agar
PEG	Polyethylene glycol
PGA	Polyglycolic acid
PLA	Poly-L-lactide acid
SEM	Scanning electron microscopy
TE	Tissue engineering
